# The Evolution of Musicality: What Can Be Learned from Language Evolution Research?

**DOI:** 10.3389/fnins.2018.00020

**Published:** 2018-02-06

**Authors:** Andrea Ravignani, Bill Thompson, Piera Filippi

**Affiliations:** ^1^Department of Language and Cognition, Max Planck Institute for Psycholinguistics, Nijmegen, Netherlands; ^2^Artificial Intelligence Lab, Vrije Universiteit Brussel, Brussels, Belgium; ^3^Research Department, Sealcentre Pieterburen, Pieterburen, Netherlands; ^4^Institute of Language, Communication and the Brain, Aix-en-Provence, France; ^5^Laboratoire Parole et Langage LPL UMR 7309, Centre National de la Recherche Scientifique, Aix-Marseille Université, Aix-en-Provence, France; ^6^Laboratoire de Psychologie Cognitive LPC UMR7290, Centre National de la Recherche Scientifique, Aix-Marseille Université, Marseille, France

**Keywords:** evolution of music, evolution of language, cultural transmission, cultural evolution, universals, music cognition, comparative cognition, nature and nurture

## Abstract

Language and music share many commonalities, both as natural phenomena and as subjects of intellectual inquiry. Rather than exhaustively reviewing these connections, we focus on potential cross-pollination of methodological inquiries and attitudes. We highlight areas in which scholarship on the evolution of language may inform the evolution of music. We focus on the value of coupled empirical and formal methodologies, and on the futility of *mysterianism*, the declining view that the nature, origins and evolution of language cannot be addressed empirically. We identify key areas in which the evolution of language as a discipline has flourished historically, and suggest ways in which these advances can be integrated into the study of the evolution of music.

## Introduction

Language and music are typical human behaviors, absent in our closest living animal relatives. Since behavior and cognition do not fossilize, the earliest stages of language and music in our ancestors can only be reconstructed indirectly. Here, we argue for the value of taking into account the evolution of language when studying the evolution of music. There are multiple reasons for this. First, from a meta-scientific perspective, the empirical investigation of language evolution predates research on the evolution of music. Second, methodologically, the fields of music and language evolution show several commonalities (Table 1). For instance, in both fields, corpus-based research is complemented by laboratory-based psychological testing, electrophysiology and neuroimaging studies, and comparative experiments on animals. Third, many hypotheses concerning the origins of music also involve language, and vice-versa. Studying language and music within a common framework provides key insights and testable hypotheses in both disciplines. Here, we argue that anti-empiricist views on language have detrimental effects on understanding its origins and evolution. We identify equivalent anti-scientific tendencies in musicology. We suggest a few ways to counteract such effects that have been established for linguistics, proposing that similar approaches should be adopted in musicology.

**Table 1:** Disciplines which can contribute to understanding the evolution of musicality, and their correspondence with affine disciplines in the evolution of language.

**Domain**	**Dimension**	**Biological constraints**	**Addressed functions**
Music	Ontogenetic development	- Organology and phoniatrics: music production- Psychoacoustics and neurophysiology: music perception- Studies on typologies and invariance of music structure: music production and perception	- Caregiver-infant emotional interaction - Prosocial behaviors
	Geo-historic development	- Geomusicology: geographic distribution of musical patterns - Archeology and ethnomusicology: historic development - Artificial Intelligence: automatic recognition and generation of musical patterns	NA
	Phylogeny	- Paleoneurology, archaeoacoustic, sensory ethnography: reconstruction of prehistoric perception of music	- Pair bonding, territorial defense, group cohesion, sexual advertisement - Emotional communication
Language	Ontogenetic development	- Psycholinguistics: speech production and comprehension, word and grammar acquisition	Communication
	Geo-historic development	Linguistics: phonology, morphology, syntax in natural languages	Communication, literature
	Phylogeny	- Evolutionary biology: mechanisms and selective pressures underlying the emergence of language	Group Identity Signaling
Common approaches to the study of music and language evolution		- Experimental Psycholinguistics and Artificial Intelligence: cultural evolution, pattern generation, semantic associations, etc.	- Emotional communication - Social interactions

Language is defined here as the ability to produce and understand verbal units within interactional communication acts. A major issue in musicology is finding an operational definition of music. Cross (2003, p.79) defines music as “embodying, entraining and transposably intentionalising time in sound and action.” Following Honing et al. (2015), we distinguish the notions of “musicality”—a set of traits which evolve as constrained by our cognitive and biological system that shape musical behaviors across cultures - and “music”—a socio-cultural artifact building on the biological inclination for musicality.

## Language and music: differences and commonalities

In language, meaningless phonemes are concatenated into larger discrete units, such as morphemes and words, in accordance with phonological and morpho-syntactic rules. These units are arbitrarily linked to meanings and conceptual representations, which may be culturally transmitted (though sound-meaning mapping in language is not always arbitrary: Monaghan et al., 2012; Parise and Spence, 2012; Imai and Kita, 2014; Dingemanse et al., 2015, 2016). In contrast, while musical tones can be concatenated into phrases or melodies according to structural rules, they are not usually arbitrarily linked to external meanings. Furthermore, while musical melodies are typically made of discrete pitches at fixed interval scales and tonal centers (in case of tonal music), spoken language involves continuous pitch rise and falls (Jackendoff, 2009).

Despite these differences, language and music share several cognitive underpinnings. A number of studies have identified common cognitive mechanisms involved in the production and perception of structural relations in both instrumental music and propositional morpho-syntax (Fedorenko et al., 2009; Patel, 2010). For instance, timing principles are used in both language and music (Ravignani et al., 2017), where longer and louder units tend to be perceived as stressed, and changes in pitch modulation orient perception of boundaries between stressed and unstressed units (Cutler et al., 1997; Curtin et al., 2005). In addition, empirical evidence from brain imaging research indicates that amusic participants show deficits in fine-grained perception of pitch (Peretz and Hyde, 2003); patients fail to distinguish a question from a statement solely on the basis of changes in pitch direction (Patel et al., 2008; Liu et al., 2010). This observed difficulty in a sample of amusic patients supports the hypothesis that music and speech intonation share specific neural resources for processing pitch patterns (but see Ayotte et al., 2002). Further brain imaging studies report a considerable overlap in the brain areas involved in the perception of pitch and rhythm patterns in words and songs (Zatorre et al., 2002; Merill et al., 2012), and in sound pattern processing in melodies and linguistic phrases (Brown et al., 2006). In adults and children, musical training facilitates syllabic and pitch processing in language (Schön et al., 2004; Besson et al., 2007).

Similarly, in both music and verbal language, emotions are expressed through similar patterns of pitch, tempo and intensity (Scherer, 1995; Juslin and Laukka, 2003; Bowling et al., 2012). For instance, in both channels, happiness is expressed by fast speech rate/tempo, medium-high voice intensity/sound level, medium-high frequency energy, high fundamental frequency (F0)/pitch level, high F0/pitch variability, rising F0/pitch contour, fast voice onsets/tone attacks (Juslin and Laukka, 2003). Importantly, the use of voice modulation to express emotional information within interpersonal communication might have had adaptive value in the early species of our genus *Homo*, improving their ability to respond appropriately to survival opportunities (Mithen, 2005; Filippi, 2016; Frijda, 2016; Filippi et al., 2017a,b).

Finally, in addition to potentially shared cognitive foundations, music and language share the feature of being socially learned from the behavioral outputs of other individuals. It is well-established that the process of transmission from one-generation to another is an important part of the cultural evolutionary process that shapes languages (Kirby et al., 2014). It is becoming clear that cultural evolutionary processes may play a similar role in shaping music (Cross, 2001, 2009; Reybrouck, 2013; Ravignani et al., 2016a; Fitch, 2017a; Ravignani and Verhoef, 2018). Cultural evolution has emerged as a unifying framework in the language sciences (Christiansen and Chater, 2008), linking the cognitive bases for language with the diversity of languages observable throughout the world. A similar approach in the study of the evolution music is desirable (Trehub, 2015).

## Research on the evolution of musicality: pitfalls to avoid

In the last decades, researchers from multiple disciplines have joined forces to unveil the origins and evolution of language. Despite patent progress in methodologies and applications (as attested by highly influential publications), a group of scientists, linguists and philosophers have strongly criticized the whole field of language evolution. This is particularly interesting because the “attack” has been led by one of the fathers of modern linguistics and cognitive science, Noam Chomsky.

A central theme of Chomsky's critiques to the empirical study of language is *mysterianism*: the idea that scientific knowledge is not always attainable (Chomsky, 2009, 2015; Piattelli-Palmarini et al., 2009). This would be due to the architecture of our minds imposing hard limits on what we can discover scientifically. In other words, Chomsky argues that the limits of human cognition hinder our capacity to unveil scientific mysteries through scientific investigation (Ravignani and Thompson, 2017). This perspective is not new, and had been already proposed in the cognitive sciences in general (McGinn, 1989).

Chomsky makes a distinction between I-language and E-language (Chomsky, 2015). I-language refers to internal linguistic representations, a universal language of thought also known as universal grammar, which, according to Chomsky, is innate. In his view, I-language has a dedicated brain area and is the only aspect of language worth studying, since it is not subject to variation through time and cultures. From his perspective, the ease with which infants acquire any of the hugely diverse set of grammatical structures observable across natural languages points to the existence of this innate universal grammar underlying language acquisition. Instead, E-language encompasses the multiple languages, i.e., the strings of sounds uttered in the outside world, which vary across individuals and cultures. Chomsky claims that processes underlying development and evolution of the wide variety of E-language instances cannot be investigated empirically. In fact, his ideas of mysterianism applied to language bring Chomsky to a simple conclusion: the most important questions about language (his I-language), including its cultural nature, its origins and development over time, and its acquisition are potentially unanswerable (Chomsky, 2015; Ravignani and Thompson, 2017). Notably, the I-language vs. E-languages distinction parallels that between *musicality*, the human cognitive-biological innate predispositions underlying music perception and production vs. *music*, intended as a cultural product (Honing et al., 2015; Honing, 2017).

Chomsky has a strong influence on debates about language evolution, and there are clear parallels between the fields of music and language evolution. Here we suggest in the strongest possible terms that research on the evolution of music should avoid Chomskyan mysterianism. By definition this perspective is scientifically stagnant, and the theoretical commitments driving Chomsky's mysterianism are widely rejected in the language sciences at large (Boeckx and Theofanopoulou, 2015; Corballis, 2017; Fitch, 2017b; Kirby, 2017; Ravignani and Thompson, 2017). Other schools of thought show some commonalities to mysterianism (McClelland, 2013; Coleman, 2016).

Another potential attack to the approach we propose comes from a field far away from Chomskyan thought. In particular, generations of ethnomusicologists and cultural anthropologists have strongly opposed the very idea of investigating evolutionary and cross-cultural features of music (Vandor, 1980; Nettl, 2005; Nattiez, 2012). These scholars often object that musical cultures from different parts of the world cannot be compared because comparison would occur through the eyes of a scholar bound to a specific culture. They argue that, across cultures, the word “music” maps to different meanings, and likewise what we call music in the Western world can be translated in many different ways across cultures and languages. According to most of these scholars, the concept of “universal”—corresponding to a feature which is found more often than not, above chance, across cultures, e.g., music often entailing percussion instruments—is pointless because it conflicts with cultural specificity. More generally, several scholars argue there is an irreconcilable divide between humanities and sciences (Gourlay, 1984; Cohen, 2001).

Both Chomskyan and anthropological schools of thought, while departing from opposite philosophical stances, reach the same theoretical conclusion: the nature of language and music is mostly unknown, and empirical efforts to unveil it are pointless. We strongly disagree with this conclusion. Even a cursory glance at the contemporary literature on language and cognition reveals astounding progress in what we know about these topics (Fitch, 2017c). Here we simply reiterate that this progress mostly results from broad contemporary adoption of the scientific method. Our view is that music-related disciplines can benefit equally from rejection of mysterianist skepticism and continued adoption of an integrated experimental approach, namely: (1) observing behaviors and the environment in which they occur, (2) formulating a hypothesis, (3) testing the hypothesis by performing an experiment or collecting new data, (4) using the results to build a model of the phenomenon of interest, (5) employing the model to generate a more refined hypothesis to be tested empirically.

Against the Chomskyan and anthropological perspectives above, we argue for an empirical approach to the origin and evolution of music and musicality. The new-born discipline of music evolution will benefit—and has already benefitted—by unifying the following approaches into one research framework: (1) empirical investigations, as opposed to armchair speculation, on ontogenetic and phylogenetic evolution of music; (2) comparative research, addressed by probing for presence of proto-musical behaviors in other animal species; (3) cross-cultural work, recognizing the diversity of world musical behaviors while comparing them to find common patterns; (4) proposing alternatives to the classical nature-nurture dichotomy. Below we discuss these four points succinctly.

## Replacing armchair speculation with models

First, centuries of scientific practice have shown that tightly integrating theory and empirics typically leads to scientific progress. The study of the evolution of music or language is not an exception to this. Theoretical frameworks *should* provide testable empirical questions (Iversen, 2016), insights for good experimental design and conceptual frameworks to interpret statistical results and generate new testable hypotheses. Ideally, theoretical contributions should be formulated as mathematical models and computer simulations. One advantage of modeling, as compared to constraint-free theorizing, is modeling's potential for falsifiability: Models rely on assumptions to make predictions, which can be empirically falsified. Another related advantage of models is their potential integration with experiments: model assumptions and predictions can be promptly translated into experimental constraints, in turn testable on humans or other living organisms. For these reasons, we strongly support the use of quantitative models of cognitive (Perfors et al., 2011; Fitch, 2014), cultural (Tamariz and Kirby, 2016), and evolutionary (Thompson et al., 2016) processes in music evolution research.

## Comparative cognition can inform human evolution

Second, the comparative approach to animal cognition can be useful in reconstructing the evolution of human behaviors. Proto-musical behaviors may emerge in other species by (1) homology, i.e. our last common ancestor with that species was endowed with a predisposition toward the behavior under scrutiny, or (2) convergent evolution, i.e., similar evolutionary pressures gave rise to similar genetic predisposition for proto-musical behaviors in humans and other species (Fuhrmann et al., 2014; Ravignani et al., 2014, 2016b; Wilson and Cook, 2016). For instance, recent studies found evidence for beat perception and production, relative pitch and tonal encoding (Hoeschele et al., 2015; Hoeschele and Bowling, 2016), octave generalization (Crickmore, 2003), and consonance (Cook and Fujisawa, 2006) in animals. Based on theoretically driven empirical research (Honing et al., 2015), we argue that, if musical tasks designed for humans are adapted - by modifying their form, not substance—to the specific species under inquiry, many “unthinkable tasks”—sensu Chomsky (2015)—may become manageable. In contrast, based on purely theoretical introspection, Chomsky argues that the cognitive differences between humans and other animals are a matter of quality, not quantity. For instance, he claims that “rats cannot deal with a prime number maze” (Chomsky, 2015; p. 105). To this, we respond that cicadas show behavioral patterns based on prime numbers (Grant, 2005; Tanaka et al., 2009); hence it is tenable that these insects could solve a cicada-adapted prime maze, say over an evolutionary timescale. Likewise, if precursors to music are studied across species with an open-minded attitude, suggestive parallels can be found across humans and other species.

## Cross-cultural comparisons

Third, an equally important sort of comparative approach consists in cross-cultural research on music. Acceptance of cross-cultural work exhibits one of the starkest contrasts between the study of language and that of music. Cross-cultural comparison of languages has proceeded unhindered for centuries, with few roadblocks. The comparative study of music, instead, experienced a golden period before a crashing halt in the 1960s. Music research can learn from evolutionary linguistics, by performing more cross-cultural work. Its purpose is to account for the uniformity and diversity of musical forms across cultures, in turn to find patterns of music and musicality which are truly generalizable to mankind (the so called universals; Savage et al., 2015).

## Beyond the nature-nurture dichotomy

Fourth, language research has been historically characterized by an overreliance on the nature-nurture dichotomy. In our view, the evolution of language as a scientific endeavor has long been plagued by a spurious dichotomy that need not be imported into the evolution of music (Fitch, 2011). We see this opposition as reflecting unnecessarily strong but historically entrenched *theoretical* divisions. On the one hand, generative linguists in the Chomskyan school have traditionally sought an exclusively mind-internal explanation for the complex tapestry of structures and operations observable in languages (although Chomsky's contemporary mysterianism questions the future of this endeavor). On the other hand, empiricists have privileged the mechanisms of human interaction and cultural transmission as explanations for the diversity and complexity of languages. These opposing standpoints have fuelled much debate but little consensus (e.g., Carruthers et al., 2005).

Contemporary nativist-empiricist dialogues in the cognitive sciences at large now focus on developing paradigms that generalize this dichotomy into a continuous range of possibilities, with traditional nativism and empiricism at the extremes. One example of such a paradigm is Bayesian cognitive modeling, in which bio-cognitive constraints and empirical learning are integrated by a theory of subjective inference based on the principles of conditional probability. The Bayesian approach provides a framework in which both empirical evidence and innate (or earlier learned) biases can be expressed as influences on how individuals learn. While earlier approaches have treated these influences as distinct alternatives, the Bayesian approach allows the two to be balanced in a formally explicit way. Several scholars have argued that approaches like this, in which any flavor of theory can be formally instantiated, interrogated, and tested against empirical data, are the future of nativist-empiricist dialogues (e.g., Spelke and Kinzler, 2009). We hope this sort of integrative approach can be imported into the evolution of music (Trehub, 2015; Jacoby and McDermott, 2017; Ravignani et al., accepted). For example, the generative program applied to music (Lerdahl and Jackendoff, 1985; Rohrmeier, 2011) has generated a range of predictions which have been tested empirically (e.g., Koelsch et al., 2013).

**Figure 1:**
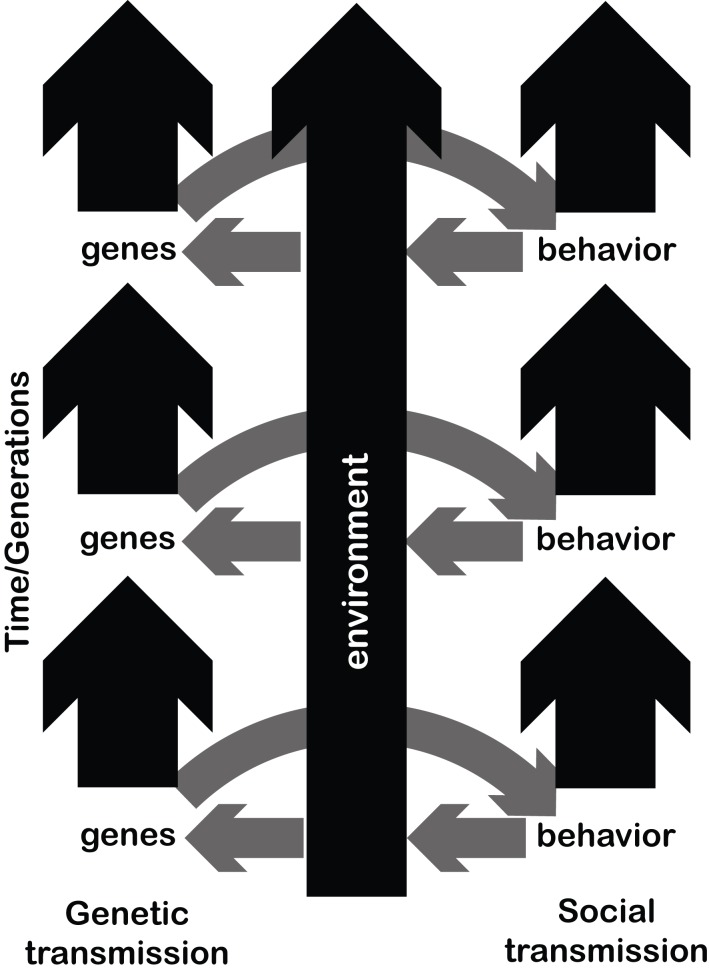
The arrows depict the potential three-way interaction between genes, environment and musical behaviors. Figure inspired by Deacon (1998). Over time, both genes **(left)** and behaviors **(right)** change and are transmitted. The environment might change stochastically, or might be directionally altered by behaviors of the species populating it. For a given generation or time period, genes affect behavioral patterns. Behavioral patterns also adapt to, and modify, their environmental medium. In turn, behaviorally-driven changes in the environment might affect the fitness landscape of a species, influencing in turn which genes will be passed on to the next generation.

In the language sciences, a new generation of researchers is pushing this approach, integrating models and empirics, even further toward larger-scale evolutionary questions (Blasi et al., 2017). This effort focuses on process-based explanations for behavior, allowing us to understand our species a unique *interaction* of bio-cognitive and cultural processes (Figure 1) via evolutionary modeling (Smith and Kirby, 2008). Modeling the evolution of minds as part of cultural systems enables us to formalize theories concerning optimal divisions of labor between specialized individual minds and the cultural processes that connect them (Thompson et al., 2016; de Boer and Thompson, 2018). Free from theoretical commitments to one evolutionary process dominating another, contemporary language scientists have at their explanatory disposal an evolutionary framework more powerful than exclusively biological or cultural explanations for behavior: co-evolution, in which evolving minds shape new behaviors, and evolving behaviors shape new minds. Like the Bayesian paradigm, co-evolutionary approaches to the origins of our abilities allow us to move past the idea that only biology or only culture is relevant to unique human behaviors, in a formally explicit way. Hence, modern approaches to the study of language evolution (e.g. Kirby et al., 2014; Thompson et al., 2016) show how biological and cognitive theory can be used to develop process-based, experimentally testable hypotheses about the emergence of behavior among culturally interacting individual minds (Trehub, 2015; Ravignani et al., accepted).

## Conclusions

We hope the evolution of music can hit the ground running under by adopting the inclusive approach described above (see Bowling et al., 2017). The main hypothesis under scrutiny is: does our species unique blend of biological and cultural features underpin remarkable human behaviors like language and music (Figure 1)?

The distinction between music and musicality provides a practical advantage in designing and interpreting experiments. Still, empirical research on the origins of music should adopt a hybrid approach, complementing experiments in tightly controlled settings—hence targeting only music or musicality—with research which integrates the two domains and explanatory levels.

Above we only describe the importance of one possible flow of ideas, from language to music. However, the inverse is also needed: More linguists would need to learn about music evolution and cognition research. For instance, phoneticians and phonologists should capitalize on music findings when investigating tone, prosody, rhythm, etc. (Brown, 2017).

In sum, recent debates provide a bird's-eye view of how the science of language has historically developed, and partly branched into the stormy study of language evolution. Scientists addressing music can benefit from historical breakthroughs and dead-ends in the study of language evolution, and use these insights to accelerate discovery in one of the most exciting topics in contemporary cognitive science, the evolution of music.

## Author contributions

All authors listed have made substantial, direct, and intellectual contributions to the work, and approved it for publication.

### Conflict of interest statement

The authors declare that the research was conducted in the absence of any commercial or financial relationships that could be construed as a potential conflict of interest.
